# The temperature variation of the CH^+^ + H reaction rate coefficients: a puzzle finally understood?†

**DOI:** 10.1039/d4cp01902d

**Published:** 2024-07-23

**Authors:** Rafael A. Jara-Toro, Octavio Roncero, François Lique

**Affiliations:** a Univ Rennes, CNRS, IPR (Institut de Physique de Rennes), UMR 6251 F-35000 Rennes France francois.lique@univ-rennes.fr; b Instituto de Física Fundamental (IFF-CSIC), C.S.I.C. Serrano 123 28006 Madrid Spain

## Abstract

CH^+^ was the first molecular ion identified in the interstellar medium and is found to be ubiquitous in interstellar clouds. However, its formation and destruction paths are not well understood, especially at low temperatures. A new theoretical approach based on the canonical variational transition state theory was used to study the H + CH^+^ reactive collisions. Rate coefficients for formation of C^+^ ions are calculated as a function of temperature. We considered the participation of a direct path and an indirect path in which the reactants should overcome an entropic barrier to form a van der Waals complex or pass through a CH_2_^+^ intermediate complex, respectively. We show that the contribution of both pathways to the formation of C^+^ has to be taken into account. The new reactive rate coefficients for the title reaction, complemented by reactive data for CH^+^/CH_2_^+^ in the H/H_2_/He mixture, have been used to simulate the corresponding kinetics experimentally measured using an Atomic Beam 22 Pole Trap apparatus at low temperature. A good agreement with the experimental findings was found at 50 K. At a lower temperature, the model overestimates the formation of C^+^. This shows that secondary reactions are not responsible for the weak C^+^ production in the experiments at such temperature. Then, we discuss the possible impact of non-adiabatic effects in the study of the H + CH^+^ reactive collisions and we found that such effects can be responsible for the decrease of the H + CH^+^ rate coefficients at low temperature. This study offers an explanation for the disagreement between H + CH^+^ theoretical and experimental rate coefficients which has been going on for 20 years and highlights the need for performing non-adiabatic studies for this simple chemical reaction.

## Abstract 2

H + CH^+^ reactive collisions are found to be not very efficient at the low temperatures that characterised the interstellar medium.

## Graphical abstract

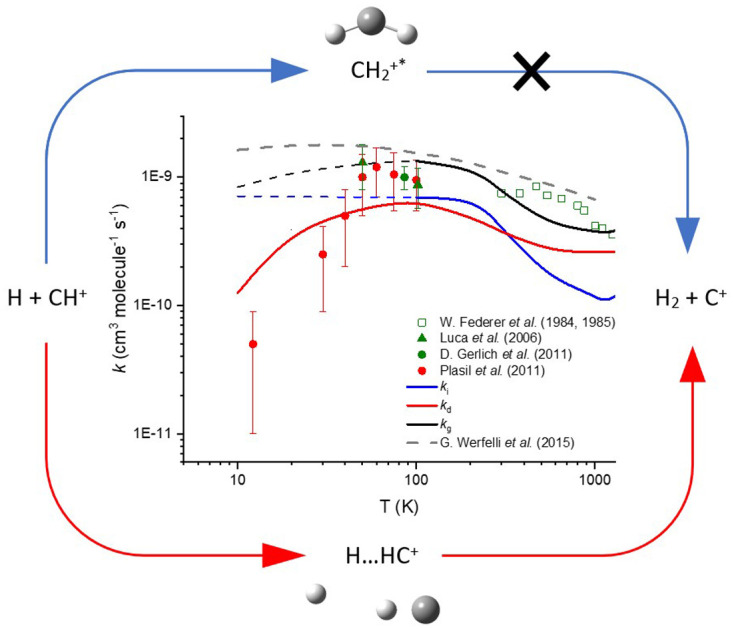

## 1. Introduction

The methylidyne cation (CH^+^) was the first molecular ion identified in the interstellar medium (ISM) in 1941.^1^ It is found to be an abundant ion in interstellar clouds. However, its relatively high abundance cannot be easily explained. Indeed, CH^+^ is found to be abundant even in the cold (neutral) ISM^2–13^ despite CH^+^ being significantly consumed by the reactions with electrons and with atomic (H) and molecular hydrogen (H_2_), the dominant constituent of the ISM. Its main formation path, through the C^+^ + H_2_ bimolecular reaction, is found to be endothermic by several thousands of Kelvins. As a result, the C^+^ + H_2_ reaction occurs only in UV irradiated regions such as photodissociation regions (PDRs) where H_2_ is radiatively pumped to excited vibrational states^14^ allowing the C^+^ + H_2_ reaction to be exothermic. Indeed, C^+^ + H_2_ (*v* = 1, 2) → CH^+^ + H quantum state-to-state rate coefficients^15–17^ used in chemical models allow the CH^+^ emission lines observed in PDRs to be reproduced well.^18,19^ For low temperature regions, where H_2_ is expected to be only in its ground vibrational state, alternative formation paths (C^+^ + H → CH^+^ + *hν*; CH + *hν* → CH^+^ + e^−^; CH_2_^+^ + *hν* → CH^+^ + H; CH_3_^+^ + *hν* → CH^+^ + H_2_; C^+^ + CH → CH^+^ + C; and C + H_3_^+^ → CH^+^ + H_2_) have been explored but their rate coefficients or probabilities to occur were found to be negligible under cold and diluted conditions that characterized the cold neutral ISM.^20^ However, we note that many of these reactions are at best only poorly known and would deserve more attention.^20^

In this context, the destruction of CH^+^ due to H collisions, a key destruction mechanism of CH^+^ in the ISM, has received considerable attention on both the experimental and theoretical sides.^19,21–32^ Despite such high interest, theoretical works failed at reproducing the experimental rate coefficients at low temperatures (below ∼60 K). The experimental studies carried out by Federer *et al.* (1984, 1985),^21,22^ Luca *et al.* (2005),^24^ and Plasil *et al.* (2011)^25^ found similar rate coefficients at temperatures above 60 K. All these experimental studies found a reactive rate coefficient of the order of 1.2 ± 0.5 × 10^−9^ cm^3^ s^−1^, in agreement with the most accurate quantum calculations and in accordance with Langevin capture theory. However, all the theoretical studies, including quantum time independent studies^29^ normally well suited for such studies, failed at reproducing the experimental measurement at low temperatures.^19,26–31,33^ Surprisingly, the experimental findings suggest a steep fall off of the reactive rate coefficients with decreasing temperature, even reaching a difference of 2 orders of magnitude with room temperature measurements, in contradiction with what is expected from simple capture models.

The most recent experimental results have been obtained using an Atomic Beam 22 Pole Trap (AB-22PT) apparatus and cover the 10–100 K temperature range.^25,34^ In the experimental device, H atoms were produced by a radio frequency (RF) discharge from H_2_ molecules so that both H and H_2_ coexist and influence the kinetics in the device. Due to the competition between reactions of CH^+^ with H atoms, H_2_ molecules and secondary reactions leading finally to CH_3_^+^ in the experimental setup, it was found to be necessary to take into account not only the diminution of the CH^+^ concentration but also the subsequent increase of the C^+^ concentration and the effective number density of H to obtain a reliable estimation of the rate coefficients for the title reaction.

The purpose of this study is to employ a new theoretical approach that allows understanding the temperature variation of the experimental rate coefficients in the 10–1000 K range and to have a better insight into the H + CH^+^ reaction mechanism. In particular, we found that correctly modeling the kinetics in the experimental device is important for a full understanding of the experimental measurements at low temperatures. Additionally, we found that non-adiabatic effects can be crucial for the theoretical study of the H + CH^+^ reaction. Indeed, by carefully modeling the kinetics of CH^+^ in the trap and by considering the possible impact of non-adiabatic effects, we were able to understand the fall off of the H + CH^+^ reaction rate coefficients and bring an explanation for the disagreement between previous theoretical and experimental studies of the H + CH^+^ reaction at low temperatures.

## 2. Computational methods

We first investigate the reaction paths of the H + CH^+^ collision using the coupled-cluster approach with single and double excitation (CCSD)^35^ along with the augmented correlation-consistent polarized valence double zeta basis set (aug-cc-pVDZ).^36^ We optimize bond distances, harmonic frequencies, and thermodynamic parameters along the minimal energy paths. The frequency analysis was used to discriminate between minima (real frequencies) or the transition state (one imaginary frequency) on the potential energy surface. For reliable energetics, the energy of each stationary point was determined with coupled cluster singles and doubles, with a perturbative treatment of triple excitation (CCSD(T))^35,36^ method and the augmented correlation-consistent polarized valence triple zeta basis set (aug-cc-pVTZ).^35,36^ All calculations were performed with the GAUSSIAN (version 16 Rev. C.01) quantum chemistry software package.^37^ The energetics of the reaction path was followed from pre-reactive stationary points considering an increment of 0.2 Å until a separation of ∼10 Å of both reactants.

The capture rate coefficients for the CH^+^ + H reaction path were calculated using the canonical variational transition state theory (CVT).^38^ This approach was chosen because the reaction bottleneck occurs at a configuration on these paths where the free energy reaches a maximum at every temperature considered in this study. For both reaction paths, we used the Rice–Ramsperger–Kassel–Marcus (RRKM) formalism implemented in the Mesmer program,^39^ to obtain microcanonical rate coefficients for dissociation of the intermediate complexes as well as for the crossing of the reaction barrier. The canonical rate coefficients for these processes were calculated by multiplying the Maxwell–Boltzmann distribution at a given temperature with the microcanonical rate coefficients at approximately the energy of the reactants for the nascent CH_2_^+^* and CH^+^⋯H intermediates on both paths (a loss of 90 cm^−1^ was considered due to the conversion of internal energy to kinetic energy). A tunneling effect was calculated using the Eckhart method.^40^

In parallel, quasi-classical trajectory (QCT) calculations have been performed with the classical molecular dynamics with the quantum transition (MDwQT) program^41,42^ on the CH_2_^+^ global potential energy surface of Stoecklin and Halvick.^26^ See Section S3 in the ESI† for a more detailed description.

## 3. Results

### 3.1. Reaction paths

**Fig. 1:**
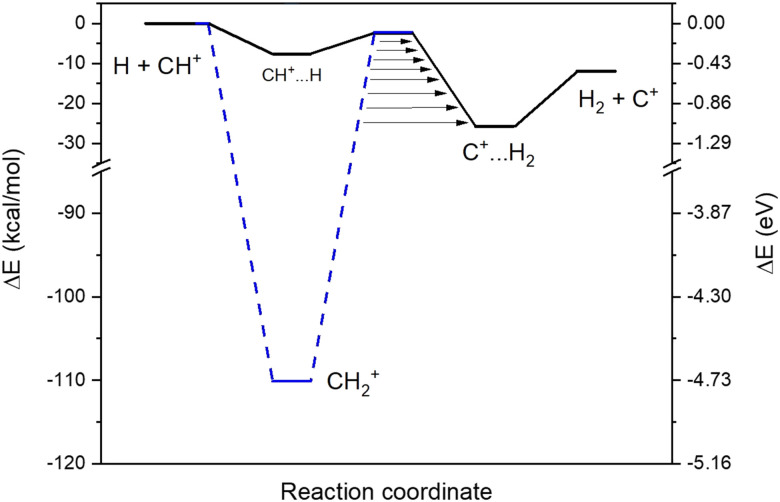
Schematic representation of the H + CH^+^ reaction. The solid black line refers to the direct path and the dashed blue line refers to the indirect path. Arrows represent the tunneling through the barriers.

We found two reaction paths following the minimal energy coordinates between the reactants (H and CH^+^) and the products (H_2_ and C^+^). They are presented in Fig. 1. The first reaction path (hereafter called an indirect path) considers the formation of the CH_2_^+^ intermediate (the blue dashed line in Fig. 1), that should, in principle, dominate the collisional process since being energetically extremely favorable. Additionally, a second reaction path (hereafter called a direct path) through a local minimum in a linear configuration (CH^+^⋯H, the black line in Fig. 1) was also found to be energetically possible even at low temperatures. Both intermediate configurations are connected to quasi iso-energetic transition states.

### 3.2. Temperature variation of the rate coefficients

In order to have a better insight into the kinetics in the experimental device, we perform a theoretical modelling of the two different reaction paths of the title reaction based on *ab initio* calculations.

For the direct path, we found that, to reach the van der Waals pre-reactive complex (CH^+^⋯H), the H has to cross positive values of the free energy in the short range. This activation barrier is induced by the repulsive force due to the collinear approach of both hydrogens and this activation barrier is leading to a decrease of the rate coefficients with decreasing temperatures. For the indirect path, for temperatures below 100 K, the free energy is always below the energy of the reactants so that the capture rate coefficients follow the Langevin capture rate coefficients.

**Fig. 2:**
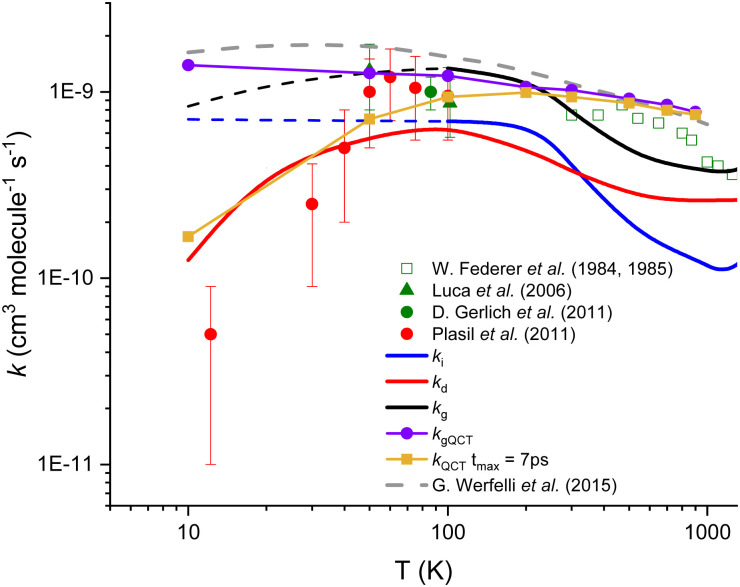
Calculated and experimental rate coefficients for the title reaction. The blue‡‡Dotted blue line is an extrapolation of the rate coefficients at low temperature due to the impossibility to converge the calculations. and red lines correspond to the present theoretical results for both indirect and direct paths, respectively, while the black line represents the global (sum of both processes) rate coefficients. Purple and gold symbols correspond to our QCT calculations. Experimental data (Plasil *et al.*^25^ (red dots), Luca *et al.*^24^ (green triangles), Gerlich *et al.*^34^ (green dot), and Federer *et al.*^21,22^ (open squares)) as well as the most accurate theoretical results of Werfelli *et al.*^29^ (grey dashed line) are presented.

In Fig. 2, the thermal rate coefficients for the two direct (*k*_d_) and indirect (*k*_i_) processes are plotted as a function of temperature. In Fig. 2, we also include the global calculated rate coefficients (*k*_g_) resulting from the sum of both processes. As one can see that our thermal rate coefficients resulting from the sum of both processes are in good agreement with the data obtained from accurate quantum scattering methods,^29^ except at very low temperature where our data are lower than the former one, the deviation being up to a factor ∼2 at 10 K. The deviation between quantum and present rate coefficients can be explained by the relatively simple approach used in this work, but also by the high difficulty of converging pure quantum reactive data for such a complex reaction, especially at low temperatures. The good overall agreement between present and state of the art calculations is however demonstrating the relative accuracy of our modeling. For the indirect process that implies the formation of a stable intermediate complex, we found that the rate coefficients are quasi-independent of the temperature up to 300 K. In contrast, for the direct path, the activation barrier generated by repulsive forces is significant at low temperatures and induces the steep fall off on the rate coefficients with a decreasing temperature.

The QCT results support the hypothesis of two competitive reactive paths, provided from the present microscopic interpretation. Indeed, the QCT rate coefficients show a near Langevin behavior and are in near quantitative agreement with the quantum results^30^ presented in Fig. 2 for temperatures above 100 K. The thermal rate coefficients were calculated under such conditions (see purple values in Fig. 2 and Fig. S3, ESI†) and are in good agreement with the Langevin limiting value and with the exact quantum results.^29^

In contrast, the analysis of the trajectories shows that below 100 K, the impact parameter increases a lot and the duration of the trajectories increases enormously (see Section S3 in the ESI†). Thus, if we consider only trajectories ending in 7 picoseconds (ps) which could be associated with the direct mechanism, the QCT rate coefficients show a decreasing behavior with decreasing temperature in good agreement with the present rate coefficients for the direct process (*k*_d_). In this view, the remaining set of trajectories, for more than 7 ps and associated with the indirect process, could give an opportunity for secondary collisions (as those described in the next section) to compete with the formation of either H_2_ + C^+^ or H + CH^+^.

### 3.3. Modelling of the kinetics in the experimental device

**Fig. 3:**
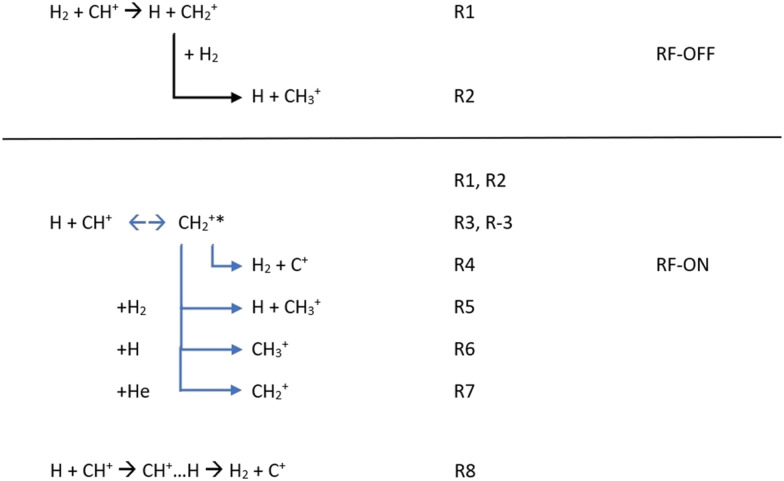
Chemical reactions possibly occurring in the experimental setup with the RF discharge set to OFF and ON.

Additionally, in order to simulate the full kinetics in the AB-22PT experimental setup used in the most recent experimental studies^25,34^ and initially composed of a mixture of CH^+^/H/H_2_/He, we decided to additionally consider all the reactions presented in Fig. 3.

If only H_2_ molecules are present in the experimental setup (*e.g.* the RF discharge is OFF), CH^+^ reacts quickly with them so that the formation of CH_2_^+^ (R1), possibly followed by a secondary reaction leading to the CH_3_^+^ product (R2), could occur. Reactions leading to more complex products such as the CH_3_^+^ + H_2_ reaction were not considered because of the absence of CH_4_^+^ or CH_5_^+^ in the experiment.^25,34,43–45^

When the RF discharge is ON (*e.g.* the discharge dissociates H_2_ into H), the previously mentioned reactions are occurring since H_2_ is still present in the device but, in addition to R1 and R2, R3 to R8 are likely to influence the kinetics of CH^+^ and as a consequence the ionic concentrations are observed. R3 to R7 are connected to the indirect path for the H + CH^+^ reaction while R8 corresponds to the H + CH^+^ direct path.

The first step for the indirect path is the formation of the CH_2_^+^* intermediate complex with an excess of internal energy (R3). This complex can (i) be destroyed to form C^+^ (R4), (ii) lead to CH_3_^+^ formation *via* secondary (R5, R6) reactions and (iii) be stabilized *via* collisions with the buffer gas (He) used to cool the stored ions in the system (R7). The formation of CH_2_^+^ in the experiment is then not only due to the reaction between CH^+^ and H_2_ (R1), but also due to the reaction between CH^+^ and H (R3 + R7).

When examining the experimentally measured concentration at temperatures of 12 and 50 K, it is obvious that the concentration of CH_2_^+^ significantly increases when the RF discharge switches from OFF to ON. However, this increase does not seem to be dependent on the temperature. This finding combined with the fact that the relative concentration of C^+^ is significantly dependent on the temperature (see Table 1) indicates that the formation of the stable CH_2_^+^ intermediate complex is not necessarily leading to the formation of C^+^ + H_2_ products. Then, it seems obvious that one has to model the full kinetics in the experimental device if one wants to accurately extract the H + CH^+^ rate coefficients.

**Table 1:** Relative experimental concentrations of CH_2_^+^ and C^+^ at different temperatures

	Relative concentrations		
	12 K	50 K (RF-ON)	50 K (RF-OFF)
CH_2_^+^	0.13^*a*^	0.14^*a*^	0.03^*a*^
	0.24^*b*^	0.21^*b*^	0.07^*b*^
	0.32^*c*^	0.30^*c*^	0.09^*c*^
C^+^	3 × 10^−4 *a*^	0.03^*a*^	—
	4 × 10^−4 *b*^	0.06^*b*^	—
	6 × 10^−4 *c*^	0.08^*c*^	—

We solved the kinetic equations with the Kintecus’ program^46^ in order to simulate the experimental concentrations of C^+^, CH^+^, CH_2_^+^ and CH_3_^+^ resulting from all the possible reactions in the experimental setup (see the ESI† for more details about the simulation).

For the rate coefficients corresponding to R1, R2, R5 and R6, we used the data provided by Plasil *et al.*^25^ and Luca *et al.*^24^ For R7, we used the collisional limit of the rigid sphere theory. More information about the values used for R1 to R8 is found in Table 2.

**Table 2:** Rate coefficients used in the kinetic model

Rate coefficients	
*k*_R1_	1.2 × 10^−9^ cm^3^ s^−1^
*k*_R2_ and *k*_R5_	1.6 × 10^−9^ cm^3^ s^−1^
*k*_R6_	1.0 × 10^−9^–1.0 × 10^−11^ cm^3^ s^−1^
*k*_R7_	3 × 10^−10^ cm^3^ s^−1^

**Fig. 4:**
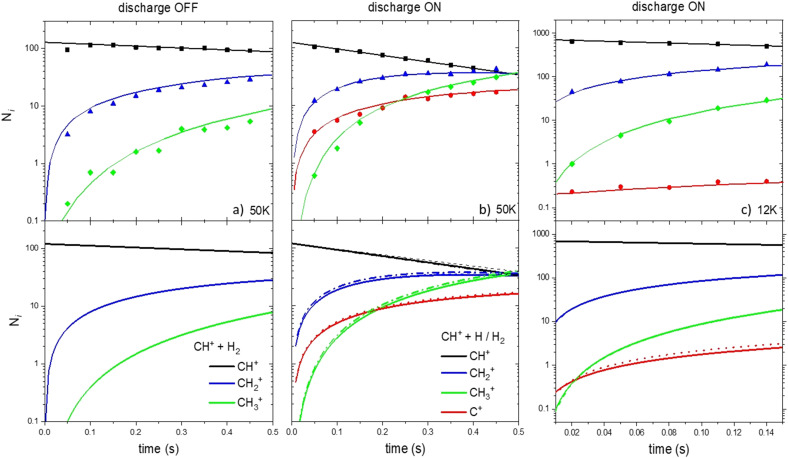
Ionic concentration as a function of the storage time. Upper panels correspond to the experimental results of Gerlich *et al.* (2001)^34^ and Plasil *et al.* (2011).^25^ Lower panels are the results from our kinetic model based on the reaction scheme of Fig. 1. Dotted and solid lines correspond to the model using set 1 and set 2 of data, respectively.

First, we investigate the experimental conditions at 50 K with the RF discharge set on OFF (only R1 and R2 occur).^24^ As shown in Fig. 4a, it is possible to reproduce very well the experimental concentrations as a function of time considering only the CH^+^ + H_2_ → CH_2_^+^ + H and CH_2_^+^ + H_2_ → CH_3_^+^ + H reactions.

Then, we simulate the concentrations of C^+^, CH^+^, CH_2_^+^ and CH_3_^+^ at 50 and 12 K but when the RF discharge is set to ON (both H and H_2_ are present in the device), two simulations were performed. In the first one, R1, R2 and only the formation of C^+^ through the H + CH^+^ reaction using *k*_g_ rate coefficients were used (set 1, dotted line). In the second one, R1 to R8 were included (set 2, solid line).

We compare the results of our simulation (using sets 1 and 2 of the data) with the results at 50 K reported by Gerlich *et al.* (2001)^34^ and Plasil *et al.* (2011)^26^ as shown in Fig. 4b. The results of the kinetic simulation using datasets 1 and 2 are in good agreement demonstrating the moderate impact of secondary events in the kinetics (the main difference between datasets 1 and 2 being the inclusion of secondary events). The agreement between the calculated and experimental C^+^, CH^+^, CH_2_^+^ and CH_3_^+^ concentrations is very good. This demonstrates that the value of the overall calculated rate coefficients for the H + CH^+^ reaction is of good accuracy.

Fig. 4c shows the results of our simulation at 12 K. The global agreement between the calculated and experimental CH^+^, CH_2_^+^ and CH_3_^+^ concentrations is good for the two datasets. However, the C^+^ concentration is overestimated by a factor 3–4 in the simulation performed with the two datasets in the kinetic model. Secondary reactions, despite being slightly more important than at 50 K, cannot explain the important decrease of the C^+^ concentration in the experimental device. Despite the deviation not being dramatic, our model fails at reproducing the experimental C^+^ concentration at 12 K and shows that secondary reactions cannot explain the deviation between theoretical and experimental results.

These simulations show that the impact of secondary reactions, despite not fully negligible, cannot explain alone the deviations between the calculated and measured H + CH^+^ reaction rate coefficients at low temperatures. Indeed, considering the lifetime of the intermediate complex, one would need a He density in the experimental setup higher by at least 3 orders of magnitude than what was considered in order to have a secondary event important. Then, one has to investigate the impact of non-adiabatic effects, neglected in the present study, if one wants to have a better insight into the full H + CH^+^ reaction mechanism. Such effects can be of high importance, especially at low temperatures, and may impact the efficiency of the title reaction.

## 4. The possible impact of non-adiabatic effects

We consider that, because CH_2_^+^* is a very stable intermediate complex with possibly a long lifetime at low temperatures, non-adiabatic processes due to the coupling between accessible electronic states by rovibronic interactions such as Renner–Teller (RT) couplings can also occur.^47^ It is known that the RT process occurs for quasi-linear configurations of the CH_2_^+^ molecule. At the linear structure, the unpaired electron of CH_2_^+^ is in one of the degenerate p-orbitals. But when the structure is slightly distorted, the unpaired electron is more likely to occupy the p-orbital parallel to the H_2_ axis, leading to a movement from the ground state to the excited state of the molecule. Non-adiabatic effects are then willing to play a major role in the calculations of the rate coefficients for the title reaction. Below, we perform a preliminary study of their possible impact.

**Fig. 5:**
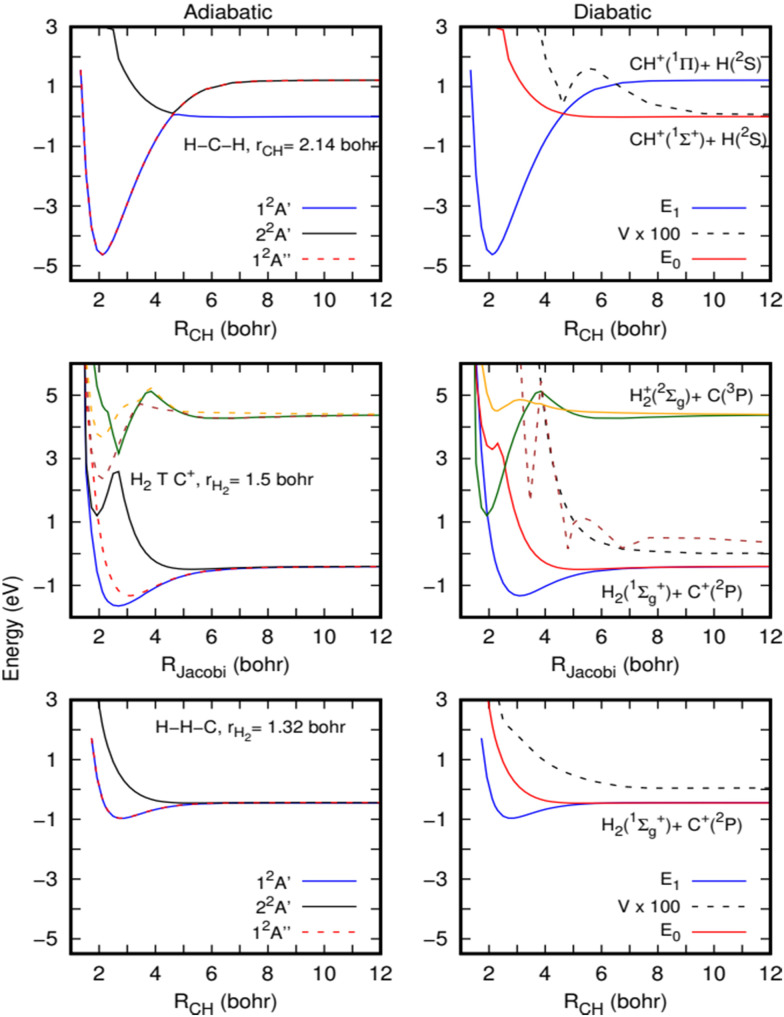
Cuts of the potential energy surface for the lower electronic states of the CH_2_^+^ system, calculated at the MRCI level (see the text for a more detailed description). Left panels correspond to adiabatic energies directly calculated (solid lines: ^2^A′ states and dashed lines: ^2^A′′ states). Right panels describe potential energies in a diabatic representation (see the text for a more detailed description). Three geometries are shown: linear HC^+^ + H (bottom panels), H_2_ + C^+^ near T-shaped geometry (89 degrees, middle panels) and linear HH + C^+^ (top panels).

In the left panels of Fig. 5, the adiabatic energies of the two lower ^2^A′ and the lower ^2^A′′ electronic states of CH_2_^+^ at three geometries are presented. The calculations have been performed with the MOLPRO suite of programs^46^ using an explicitly correlated internally contracted multi-reference configuration interaction (ic-MRCI-F12)^48–50^ and the cc-pCVTZ-F12 electronic basis set.^51^ The molecular orbitals are optimized using the state-averaged complete active space self-consistent field (SA-CASSCF) method, with 6 active orbitals and considering 6 electronic states (3A′ and 3A′′).

For the H–C–H configuration (top panels of Fig. 5), there is a clear crossing between the two A′ curves, which gives rise to a shallow barrier at this collinear geometry. As discussed by Stoecklin and Halvick,^26^ CH^+^ in its ground electronic state (X^1^Σ^+^) has no electron available for bonding and its interaction with the hydrogen atom gives rise to a repulsive curve. However, the CH^+^ in its first excited electronic state (A^1^Π) forms a deep well at this geometry. The curves of these two states cross at a CH distance of approximately 2.5 Å, giving rise to the ground electronic state potential with a small barrier.

The top of this barrier at collinear geometry corresponds to a conical intersection (CI) and clearly suggests the possibility of non-adiabatic transitions towards the excited ^2^A′ state. To illustrate this further, diabatization has been performed using the method described in ref. 52. The diabatic curves are shown in the right panels of Fig. 5. Using such a method, an electronic subspace of three states is considered, formed by doubly degenerate Π electronic states and one Σ electronic state, corresponding to *E*_1_ and *E*_0_ in the figure. The Σ electronic state is coupled to the two Π electronic states, by the coupling *V*, but there is no direct coupling between the two Π electronic states. As expected, the *E*_0_ is repulsive while the two *E*_1_ form a deep well and there is a non-negligible coupling among them (it should be noticed that the calculations are done at an angle of 179.99 degrees, and this is the reason for the non-zero coupling). A very similar situation holds for other angles where the crossings are avoided because the couplings are much stronger.

The barrier originated by this CI was included in the fits made for the ground adiabatic electronic state,^26,28^ and accurate quantum calculations performed on these PESs^29–31^ did not show any decrease in the H + CH^+^ → H_2_ + C^+^ reactive rate coefficients. Such behaviour is also supported by the present QCT calculations (see the ESI†), which show a near Langevin behaviour as the quantum calculations,^29^ and we may then conclude that the cusp introduced by the CI in the ground electronic state is not able to reduce the reactivity.

However, near conical intersections, the flux can bifurcate among the different electronic states involved in the crossing, and we therefore expect some reduction in the reactive rate coefficients when including electronic transitions. It is also interesting to note that the non-adiabatic diagonal terms, proportional to the second derivatives of the electronic functions with respect to nuclear coordinates, typically include an additional barrier, which could yield a partial reflection back, thus reducing the reaction rate coefficients.

The complex non-adiabatic character appearing in the CH_2_^+^ complex is further illustrated in the middle panels of Fig. 5, where cuts of the first six electronic states (3^2^A′ and 3^2^A′′) are shown for a T-shaped geometry (in fact 89 degrees to keep the *C*_s_ symmetry), for a slightly elongated H_2_ (at *r* = 1.51 Bohr, *r* being the H–H distance). The upper three electronic states correlate with the C(^3^P) + H_2_^+^ (X^2^Σ) asymptote, leading to two states out of the plane and a state in the plane of the molecule. These three states show a slight energy increase of energy between 4 and 6 Bohrs, and at shorter distances they show a sudden energy decrease, due to crossings with other even more excited Rydberg states. As the H_2_ internuclear distance increases and the C^+^ insets in the middle, the well appearing in the 2^2^A′ state, at *R* = 2 Bohr (*R* is the distance between C^+^ and the center of the mass of H_2_) and 1 eV, gets deeper, being responsible for the deep well of the CH_2_^+^ ground electronic state. Another well is also apparent at this geometry for the 3^2^A′′ state, which give rises to the second Π electronic state originating from the Renner–Teller effect in CH_2_^+^.

This situation was also described in ref. 47, and the CI appears in this case for T-shaped geometries. The crossings are also illustrated in the diabatic representation shown in the right-middle panel of Fig. 5. All these show that below the H + CH^+^ asymptote there are several electronic states which are strongly coupled. Thus, strong non-adiabatic transitions are expected to occur, which may reduce the reaction probability.

In the H–H–C geometry shown in the top panels of Fig. 5, in the exit channel towards the H_2_ (X^1^Σ^+^) + C^+^(^2^P) asymptote, the lower eigenvalues correspond to the doubly degenerate Π electronic states, and a similar situation holds for other bent geometries.

**Fig. 6:**
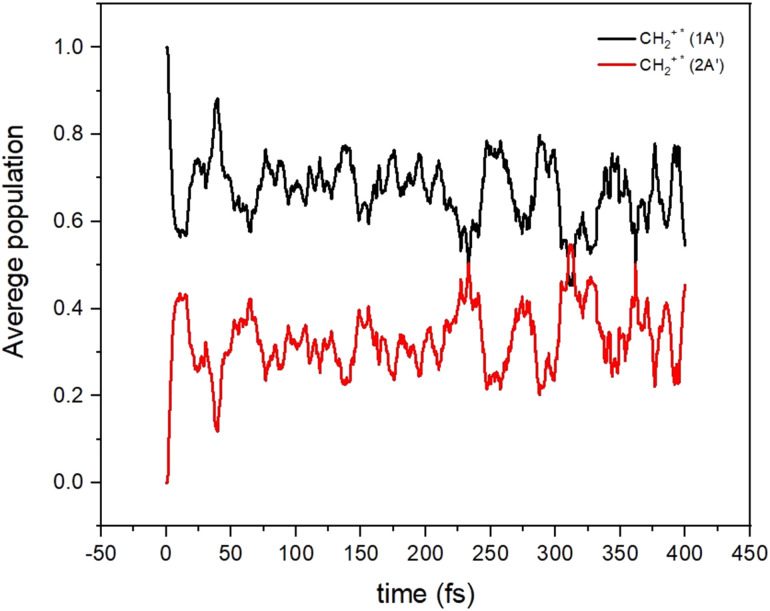
Average populations of the ground (1A′) and first excited (2A′) electronic states for CH_2_^+^* as a function of time.

All these results indicate the possibility that non-adiabatic transitions among these electronic states are indeed possible and should have an effect on the dynamical calculations. To show this, we have performed on-the-fly classical trajectories using Tully's method and a lower level of theory, a time-dependent density functional theory (TD-DFT). 100 non-adiabatic dynamics were made (see Fig. 6) starting from the CH_2_^+^* molecule (the CH_2_^+^* molecule has an energy corresponding to that of the reactants). The non-adiabatic dynamics were carried out with the Newton-X program interfaced with the GAUSSIAN (version 16 Rev. C.01) using the wb97xd/aug-cc-pvdz with the long-range and dispersion corrected level of theory.^53^ Such a method was selected because of its relative accuracy and especially its numerical efficiency since multiple simulations of the dynamics were required for high confidence in the results. The accuracy of this approach *versus* that of highly correlated quantum chemistry approaches was tested (see Section S4 in the ESI†) and we found that this level of theory can provide a good representation of the electronic energies and structure of the collisional complex.

It can be seen that the population initially in the 1^2^A′ state is transferred very quickly to the 2^2^A′ state, where it remains for a long time. The increase of the density of vibronic states, because of the many electronic states participating in the dynamics, may induce the increase of the life-time of the CH_2_^+^* intermediate complex. Under such a situation, the long-lived CH_2_^+^* complex could have more time to suffer other relaxing processes (R5–R7 in Fig. 2), thus reducing the C^+^ channel. Also, the crossings among electronic states of the ionic character could also lead to the increase of diagonal and non-diagonal transition dipole moments which could accelerate to some extent the radiative emission to form CH_2_^+^.

## 5. Conclusions

This study clearly demonstrates that non-adiabatic effects can significantly influence the modelling of the title reaction. Indeed, the magnitude of the rate coefficients for the title reaction can be significantly different if a non-Born–Oppenheimer approach including non-adiabatic effects is considered as already found for example for the F + H_2_ reaction.^54^

Then, it seems crucial to perform a non-adiabatic study of the H + CH^+^ reaction involving multiple potential energy surfaces and couplings between them as emphasised by the authors of experimental studies on the title system. Indeed, the indirect path considered in most of the theoretical studies involves a Langevin reaction which only occurs under adiabatic conditions. The present study is the first one allowing qualitative and (in part) quantitative analyses of the CH^+^ + H experiments at low temperatures. It is indeed offering an alternative to the persisting disagreement between the experimental and theoretical studies for the title reaction. It is now obvious that a non-adiabatic study of the reaction has to be performed in order to improve our knowledge of the reaction and possibly for the theory to match the experimental data.

From the astrophysical point of view, the decrease of the rate coefficients at low temperatures is consistent with the relatively high abundance of CH^+^ in cold astrophysical media. Indeed, the use of much lower rate coefficients for the H + CH^+^ reaction than those actually used (∼10^−9^ cm^3^ s^−1^, see the KIDA database^55^) would help in reconciliating observations of CH^+^ and astrochemical models. We also highlight that new experimental studies at low temperature using an alternative experimental setup such as molecular beams would be very valuable in order to better know and understand the peculiar reaction mechanism of the H + CH^+^ reaction.

## Data availability

The data supporting this article have been included as part of the ESI.†

## Conflicts of interest

There are no conflicts to declare.

## Supplementary Material

CP-026-D4CP01902D-s001
