# Supplementary material for: The temperature variation of the CH+ + H reaction rate coefficients: a puzzle finally understood?
Source: Phys Chem Chem Phys. 2024 Jul 23;26(32):21370–8. doi: 10.1039/d4cp01902d (PMC11323935; doi:10.1039/d4cp01902d)
Supplement: CP-026-D4CP01902D-s001 [file CP-026-D4CP01902D-s001.pdf]

Supplementary Information (SI) for Physical Chemistry Chemical Physics.
This journal is © the Owner Societies 2024

The temperature variation of the CH^+ + H reaction rate coefficients: a puzzle finally understood?

Rafael A. Jara-Toro[1], Octavio Roncero[2] and François Lique[1*]

1. Univ Rennes, CNRS, IPR (Institut de Physique de Rennes) - UMR 6251, F-35000 Rennes, France.
2. Instituto de Física Fundamental (IFF-CSIC), C.S.I.C., Serrano 123, 28006 Madrid, Spain.

E-mail: francois.lique@univ-rennes.fr

S1. Calculated rate coefficients

Table S1. Calculated rate coefficients.

	Indirect path				Direct path	
T (K)	k_{R3} (cm^3 s^{-1})	k_{R-3} (s^{-1})	k_{R4} (s^{-1})	k_i (cm^3 s^{-1})	$k_{d\ or\ R9}$ (cm^3 s^{-1})	K_G (CM^3 S^{-1})
**10**	----	----	----	$7.11x10^{-10}$ *	$1.25x10^{-10}$	$8.36x10^{-10}$ *
**20**	----	----	----	$7.05x10^{-10}$ *	$3.91x10^{-10}$	$1.10x10^{-9}$ *
**50**	----	----	----	$7.00x10^{-10}$ *	$5.71x10^{-10}$	$1.27x10^{-9}$ *
**70**	----	----	----	$6.97x10^{-10}$ *	$6.17x10^{-10}$	$1.31x10^{-9}$ *
**100**	$8.93x10^{-10}$	$3.91x10^{9}$	$1.37x10^{10}$	$6.95x10^{-10}$	$6.44x10^{-10}$	$1.34x10^{-9}$
**150**	$9.53x10^{-10}$	$5.40x10^{9}$	$1.39x10^{10}$	$6.86x10^{-10}$	$5.55x10^{-10}$	$1.24x10^{-9}$
**200**	$9.66x10^{-10}$	$7.23x10^{9}$	$1.43x10^{10}$	$6.42x10^{-10}$	$4.85x10^{-10}$	$1.13x10^{-9}$
**250**	$8.81x10^{-10}$	$9.35x10^{9}$	$1.48x10^{10}$	$5.40x10^{-10}$	$4.27x10^{-10}$	$9.67x10^{-10}$
**298**	$7.87x10^{-10}$	$1.17x10^{10}$	$1.52x10^{10}$	$4.45x10^{-10}$	$3.76x10^{-10}$	$8.21x10^{-10}$
**500**	$4.61x10^{-10}$	$2.42x10^{10}$	$1.71x10^{10}$	$1.91x10^{-10}$	$2.84x10^{-10}$	$4.75x10^{-10}$
**700**	$4.57x10^{-10}$	$4.09x10^{10}$	$1.91x10^{10}$	$1.45x10^{-10}$	$2.63x10^{-10}$	$4.08x10^{-10}$
**1000**	$5.04x10^{-10}$	$7.38x10^{10}$	$2.25x10^{10}$	$1.18x10^{-10}$	$2.60x10^{-10}$	$3.78x10^{-10}$
**1100**	$5.23x10^{-10}$	$8.67x10^{10}$	$2.37x10^{10}$	$1.08x10^{-10}$	$2.63x10^{-10}$	$3.71x10^{-10}$
**2000**	$4.50x10^{-9}$	$2.46x10^{11}$	$3.73x10^{10}$	$5.92x10^{-10}$	$3.17x10^{-10}$	$9.09x10^{-10}$

* These rate coefficients were obtained from the tendency of the other k_i and are shown as dashed line in Figure 2. Their microcanonical rate coefficients could be calculated due to the impossibility of reaching convergence.

Table S2. *Rate coefficients used in the kinetic model*

Rate coefficients	
k_{R1}	$1.2x10^{-9}$ cm^3 s^{-1}
k_{R2} **and** k_{R5}	$1.6x10^{-9}$ cm^3 s^{-1}
k_{R6}	$1.0x10^{-9}$ - $1.0x10^{-11}$ cm^3 s^{-1}
k_{R7}	$3x10^{-10}$ cm^3 s^{-1}

k_{R1}, k_{R2} and k_{R5} were taken from ref 1.
A range of rate coefficients was used for k_{R6} without any effect on the global concentrations.
For $k_{R7,}$ was used the collisional limit for the rigid sphere theory.

S2. Kinetic modelling

The CH^+ + H reaction was modeled assuming steady-state conditions and solving the kinetic equations as a function of time and using the initial reactant concentrations.

RF-OFF kinetic modelling at 50K

For the reactive process without discharge, the CH^+, H_2, CH_2^+, CH_3^+, and He concentrations were settled at 120 cm^{-3}, 6×10^8 cm^{-3}, 0 cm^{-3}, 0 cm^{-3}, and 3.7×10^{12} cm^{-3}, respectively.

RF-ON kinetic modelling at 50K

The CH^+, H_2, H, CH_2^+, CH_3^+, and He concentrations taking into account the discharge ON were settled at 120 cm^{-3}, 1.7×10^9 cm^{-3}, 4×10^8 cm^{-3}, 0 cm^{-3}, 0 cm^{-3}, and 3.7×10^{12} cm^{-3}, respectively.

RF-ON kinetic modelling at 12K

The CH^+, H_2, H, CH_2^+, CH_3^+, and He concentrations taking into account the discharge ON were settled at 700 cm^{-3}, 1.2×10^9 cm^{-3}, 4×10^7 cm^{-3}, 0 cm^{-3}, 0 cm^{-3}, and 1.5×10^{13} cm^{-3}, respectively.

S3. Quasi-classical trajectory (QCT) dynamics

For the QCT calculations, trajectories were made with the classical molecular dynamics with quantum transition (MDwQT) program[2-3] on the potential energy surface of Stoecklin and Halvick[4]. First, 10000 trajectories were running with the maximum impact parameter settled at 10 bohrs and dynamics were stopped when the distance between the atoms is larger than 22 bohrs. The results are showed in Figure S1.

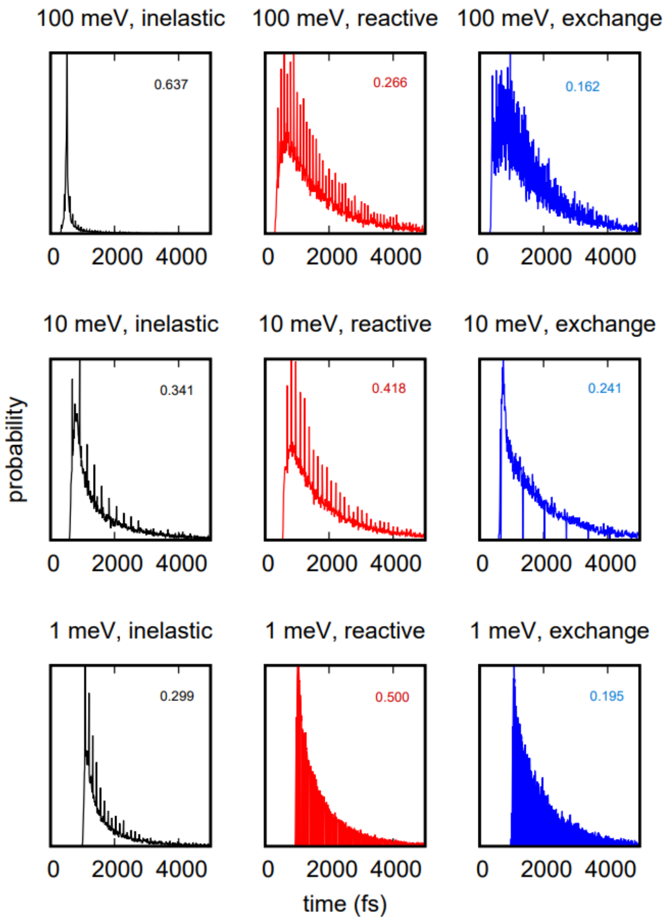

Figure S1. *Various processes probability for the CH^+ + H collision as a function of time at different kinetic energies. The numbers inside each plot are the observed fraction of the process.*

From these results, it can be seen that the reactive process is dominant at low energies but in competition with the inelastic process (dissociation of the CH_2^{+*} into the product). This is consistent with the microcanonical rate coefficients computed in this work.

By analyzing the reaction probabilities, we can observe distinct patterns. At high energies, the probability for the consumption of the pre-reactive complex (CH_2^{+*} or CH^+…H) is a Boltzmann function. Initially, low reaction probabilities are found. Then, it is followed by a gradual increase before a constant decrease. This behavior aligns with the presence of both direct and indirect reaction paths. At low energy, the probabilities are rapidly increasing and then suddenly decreasing. This behavior suggests that the indirect path is strongly dominant under adiabatic conditions. Such finding is consistent with the most

accurate quantum calculations where the collision process occurs through global minimum of the ground state of CH_2^+ previous to the $C^+ + H_2$ formation.[5-12]

Additionally, $5x10^5$ trajectories were performed for 10 K and 50 K and $1x10^6$ trajectories for temperatures above 100 K (Figure S2). The maximum impact parameter used for all the trajectories was 40 bohrs. Figure S2 indicates that when the temperature is decreasing, larger impact parameters play an increasingly important role. Such large impact parameters correspond to very long (in time) trajectories that lead to spiral-shaped orbits before reacting.

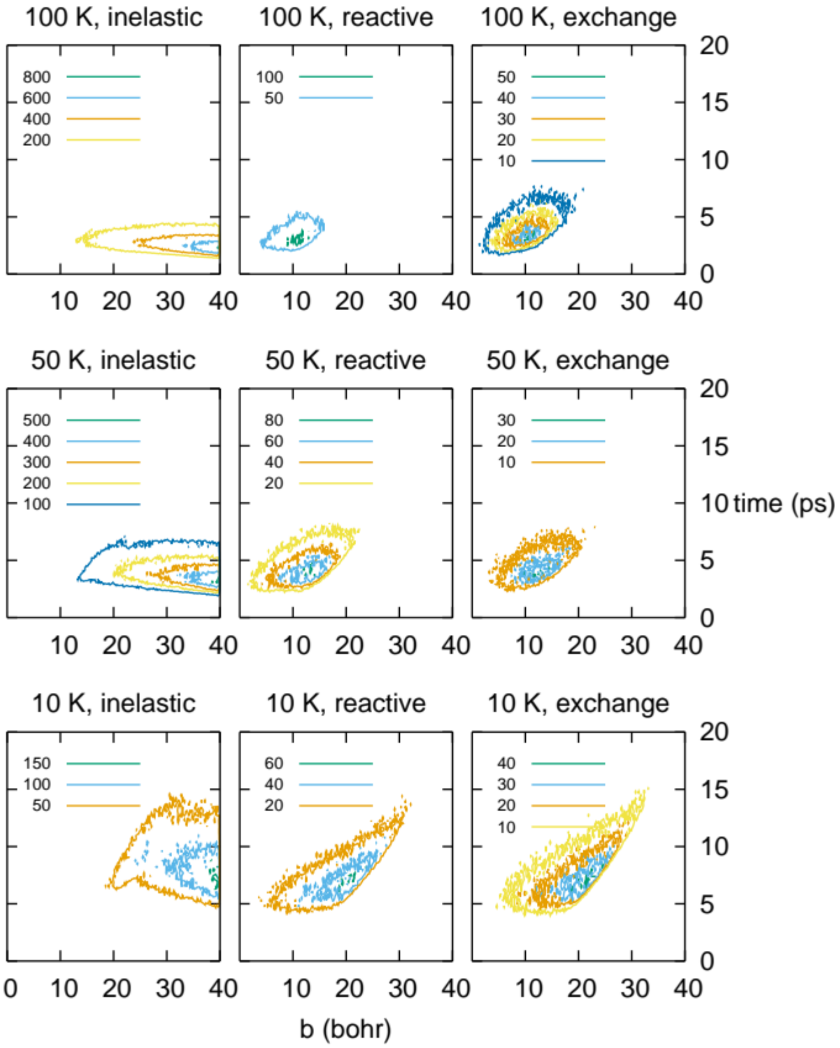

Figure S2. *Contour plot of the number of trajectories as a function of the impact parameter and total time of the trajectories.*

The thermal rate coefficients were calculated using such conditions (see purple values in Figure S3) and are in good agreement with the Langevin limiting value and with the exact quantum results[7] ($1.38x10^{-9}$ cm³ s^{-1}, $1.26x10^{-9}$ cm³ s^{-1} and $1.22x10^{-9}$ cm³ s^{-1}, respectively).

However, we observe in Figure S3 that when the maximum time allowed for the reactive process is reduced, the thermal rate coefficients are also reduced, in a nearly quantitative agreement with the results of the present microcanonical model. Thus, longer time trajectories may yield to secondary collisions or to other phenomena, as those labelled as R5-R7 in the main text. This microscopical dynamical picture can be used to justify the direct (fast) and indirect (slow) mechanism allowing to produce only a gradual decay of the reactive rate coefficients when reducing temperature.

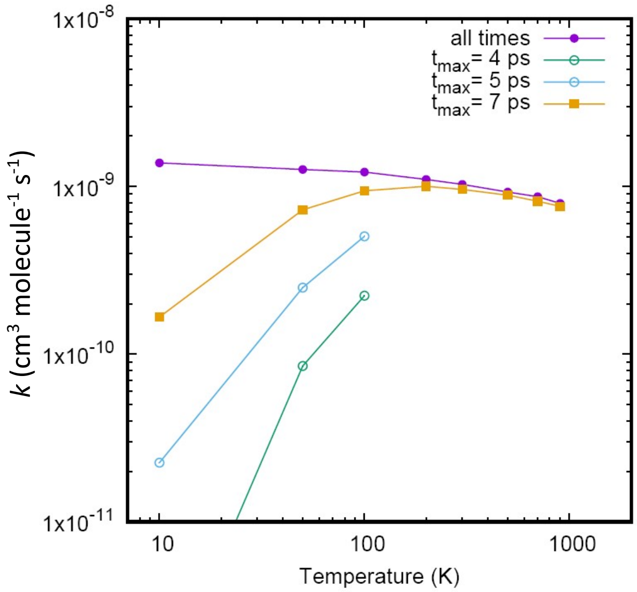

Figure S3. *Effect of the maximum time allowed for the reactive process on the thermal rate coefficients*

S4. Benchmark between wb97xd/aug-cc-pvdz method vs. high level calculations.

The wb97xd/aug-cc-pvdz level of theory used for the non-adiabatic molecular dynamics is benchmarked versus experimental and theoretical values of reference. We computed the equilibrium geometry of CH_2^+ in both its ground and first excited electronic states and compare our results to those obtained by MRCI and CCSD(T) calculations of reference and to experimental measurement. The comparison is shown in Table S3.

Table S3. *Comparison of the equilibrium geometry of* CH_2^+ *in the ground and first excited electronic states calculated at the* wb97xd/aug-cc-pvdz level of theory *with data available in the literature. Unit of R1 (H-H distance), R2 (C-H1 distance) and R3 (C-H2 distance) is* a_0 *and unit of Θ (H-C-H angle) is deg.*

Feature	R1	R2	R3	Θ
**Global minimum (ground electronic state)**				
**This work (wb97xd/AVDZ)**	3.937	2.091	2.091	140.6
**Ref. 13 (MRCI(Q)/AV5Z and VQZ)**	3.859	2.063	2.063	138.6
**Ref. 12 (MRCI(Q)/AV6Z)**	3.896	2.067	2.067	141.0
**Ref. 14 (CCSD(T)/aug-cc-pVXZ, X= Q and 5)**	3.890	2.067	2.067	140.39
**Ref. 15 (exp.)**	3.922	2.088	2.088	139.8
**Global minimum (first excited state)**				
**This work (wb97xd /AVDZ)**	4.160	2.081	2.081	180.0
**Ref. 16 (MRCI(Q)/AV6Z)**	4.118	2.059	2.059	180.0

The similarities between the results obtained with the different level of theory and the experimental data show that wb97xd/aug-cc-pvdz level of theory used in this work can provide a good representation of the geometrical evolution in the regions around the CH_2^+ and the Renner-Teller crossing using the on-the-fly dynamics.

References

1 Gerlich, D., Borodi, G., Luca, A., Mogo, C. & Smith, M. A. Reactions between cold CH_x^+ and slow H and H_2. *Zeitschrift fur Physikalische Chemie* **225**, 475–492 (2011).

2 Zanchet, A., Roncero, O. & Bulut, N. Quantum and quasi-classical calculations for the S+ + H2 (v,j) → SH+ (v',j') + H reactive collisions. *Phys. Chem. Chem. Phys.* 18, 11391–11400 (2016).

3 Sanz-Sanz, C., Aguado, A., Roncero, O. & Naumkin, F. Non-adiabatic couplings and dynamics in proton transfer reactions of H_n^+ systems: Application to $H_2+H_2^+ \rightarrow H+H_3^+$ collisions. *J. Chem. Phys.* 143, 234303 (2015).

4 Stoecklin, T. & Halvick, P. Low temperature quantum rate coefficient of the H + CH^+ reaction. *Phys. Chem. Chem. Phys* **2**, 2446–2452 (2005).

5 Sundaram, P., Manivannan, V. & Padmanaban, R. Dynamics and resonances of the H(^2S) + $CH^+(X^1\Sigma^+)$ reaction in the electronic ground state: A detailed quantum wavepacket study. *Phys. Chem. Chem. Phys.* **19**, 20172–20187 (2017).

6 Bonfanti, M., Tantardini, G. F. & Martinazzo, R. Adiabatic potential energy surfaces for the low-energy collisional dynamics of $C^+(^2P)$ ions with H_2 molecules. *J. Phys. Chem. A* **118**, 6595–6603 (2014).

7 Werfelli, G., Halvick, P., Honvault, P., Kerkeni, B. & Stoecklin, T. Low temperature rate coefficients of the H + $CH^+ \rightarrow C^+$ + H_2 reaction: New potential energy surface and time-independent quantum scattering. *J. Chem. Phys.* **143**, 114304 (2015).

8 Halvick, P., Stoecklin, T., Larrégaray, P. & Bonnet, L. Cross sections and low temperature rate coefficients for the H + CH^+ reaction: A quasiclassical trajectory study. *Phys. Chem. Chem. Phys* **9**, 582–590 (2007).

9 Warmbier, R. & Schneider, R. Ab initio potential energy surface of $CH^+{}_2$ and reaction dynamics of H + CH^+. *Phys. Chem. Chem. Phys* **13**, 10285–10294 (2011).

10 Bovino, S., Grassi, T. & Gianturco, F. A. CH+ Destruction by Reaction with H: Computing Quantum Rates to Model Different Molecular Regions in the Interstellar Medium. *J. Phys. Chem. A*, **119**, 11973–11982 (2015).

11 Faure, A. *et al.* State-to-state chemistry and rotational excitation of CH^+ in photon-dominated regions. *Mon. Not. R. Astron. Soc.*, **469**, 612–620, (2017).

12 Li, Y. Q., Zhang, P. Y. & Han, K. L. Accurate high level *ab initio* -based global potential energy surface and dynamics calculations for ground state of CH_2^+. *J. Chem. Phys.*, **142**, 124302, (2015).

13 L. Guo, H. Ma, L. Zhang, Y. Song and Y. Li, Accurate global potential energy surface for the ground state of CH_2^+ by extrapolation to the complete basis set limit, *RSC Adv.*, **8**, 13635–13642, (2018).

14 N. R. Brinkmann, N. A. Richardson, S. S. Wesolowski, Y. Yamaguchi and H. F. Schaefer, Characterization of the $\tilde{X}^2A_1$ and a^4A_2 electronic states of CH_2^+, *Chem. Phys. Lett.*, **352**, 505–510, (2002).

15 S. Willitsch and F. Merkt, Characterization of the $\tilde{X}^2A_1$ (0,0,0) ground vibronic state of CH_2^+ by pulsed-field-ionization zero-kinetic-energy photoelectron spectroscopy, *J. Chem. Phys.*, **118**, 2235–2241, (2003).

16 H. Ma, C. Zhang, Y. Song, F. Ma and Y. Li, Accurate High-Level Ab Initio-Based Global Potential Energy Surface and Quantum Dynamics Calculation for the First Excited State of CH_2^+, *J. Phys. Chem. A*, **125**, 5490–5498, (2021).
